# Microwave Hydrodiffusion and Gravity (MHG) Extraction from Different Raw Materials with Cosmetic Applications

**DOI:** 10.3390/molecules25010092

**Published:** 2019-12-25

**Authors:** Lucía López-Hortas, Elena Falqué, Herminia Domínguez, María Dolores Torres

**Affiliations:** 1 Department of Chemical Engineering, Faculty of Sciences, University of Vigo, Edificio Politécnico, As Lagoas s/n, 32004 Ourense, Spain; luloho@gmail.com (L.L.-H.); herminia@uvigo.es (H.D.); 2 Department of Analytical Chemistry, Faculty of Sciences, University of Vigo, Edificio Politécnico, As Lagoas s/n, 32004 Ourense, Spain; efalque@uvigo.es

**Keywords:** *Cytisus scoparius*, *Pleurotus ostreatus*, *Brassica rapa*, *Quercus robur*, sun creams, thermal spring waters

## Abstract

Microwave hydrodiffusion and gravity (MHG) and ethanolic solid-liquid extraction were compared using selected plant sources. Their bioactive profile, color features, and proximate chemical characterization were determined. MHG extracts, commercial antioxidants, and three distinct types of thermal spring water were used in a sunscreen cream formulation. Their bioactive capacity, chemical and rheological properties were evaluated. MHG *Cytisus scoparius* flower extract provided the highest bioactive properties. *Pleurotus ostreatus* MHG liquor exhibited the highest total solid extraction yield. The *Brassica rapa* MHG sample stood out for its total protein content and its monosaccharide and oligosaccharide concentration. *Quercus robur* acorns divided into quarters supplied MHG extract with the lowest energy requirements, highest DPPH inhibition percentage, total lipid content and the highest enzyme inhibition. The chemical and bioactive capacities stability of the sunscreen creams elaborated with the selected MHG extracts and the thermal spring waters showed a similar behavior than the samples containing commercial antioxidants.

## 1. Introduction

In the last decade, cosmetics from natural botanical sources have gained increasing interest due to their healthy features avoiding synthetic components in the matrices [1,2]. The consumer demand has notably grown due to the rise of sensitive skin, atopic diseases or allergies. In this field, spring waters can be used in different treatments and rehabilitation of patients in the hydrotherapy context. The positive effect of the hot spring water therapy in functional improvements, musculoskeletal diseases, and rehabilitation of patients in sports medicine is well known [3]. Recent studies have indicated that the physical properties and chemical effects of this type of water have a potential application in immuno-inflammatory processes, chronic pain diseases, chronic cardiac procedures, metabolic syndromes and neurological illnesses [4]. The use of thermal waters in dermatology treatments provides therapeutic benefits due to their non-pathogenic microbes and mineral content. The use of different topical products made with thermal waters enhances several dermatitis diseases [5,6,7].

The natural bioactive compounds used in cosmetics can provide a variety of activities, acting as humectants, photoprotective, antioxidant, antimicrobial, or anti-aging agents [8,9,10]. Alternative botanical sources of interest could be employed in cosmetic products since numerous bioactive compounds with health benefits in this sense derived from vegetable raw materials. In this work, some undervalued wild resources and agriculture crops were used to recover extracts with probable use in cosmetic products. Extracts of *Cytisus scoparius* flowers were employed in a sunscreen cream since this plant contains polyphenolic compounds with antioxidant and antibacterial capacities without skin irritation response [11,12]. *Pleurotus ostreatus* is a mushroom widely used in cosmeceuticals products. This cosmetic ingredient offers antioxidant, antimicrobial and anti-tyrosinase activities [13,14]. *Brassica rapa* tap root extracts also have a potential cosmetic use since its tyrosinase enzymatic inhibition promotes the skin depigmentation [15,16]. For this reason, the authors consider the evaluation of the *Brassica rapa* var. *rapa* leaves extract since this crop is a traditional and extensive plant produced in Galicia area (northwest of Spain). *Quercus robur* is a tree very abundant in the Atlantic forest. Their acorns presented a noticeable content of antioxidant and phenolic compounds that can be very useful in cosmetic products [17,18]. It should be highlighted that despite the number of natural cosmetic applications, further scientific studies with comprehensive physicochemical and mechanical analysis of the natural sunscreen creams are required in order to improve the processing, storage and quality of the end product.

The use of environmentally friendly methods for the extraction of the functional ingredients of cosmetics has been another challenge in recent years [19]. Microwave hydrodiffusion and gravity (MHG) could be a solvent-free extraction attractive alternative to obtain bioactive fractions from natural resources in a sustainable manner [20] in contrast with the conventional solid-liquid extraction (SLE) technique. For example, *C. scoparius* branches were extracted with *n*-hexane solvent in a Soxhlet extractor [11]. These types of extraction procedures demand important volumes of solvents with high purity and extensive time extraction period which means that these processes are expensive and imply problems associated with the toxic danger features of the used solvents for foodstuff, pharmacology and cosmetic uses [21].

In this context, the aim of this paper was the employment of MHG technology with different agroforestry raw materials for their valorization as a source of high value-added antioxidant compounds. Yields and energy consumptions were evaluated. Total phenolic content, antioxidant capacity, total carotenoid content and capacity of anti-elastase and -tyrosinase enzymatic inhibition of the collected liquid phases by this innovative extraction method were evaluated. Principal macronutrients present in these samples as well as their color features were also examined. These properties were also defined for the recovered solid phases. This study was complemented with the incorporation of the selected extracts in a sun cream product formulated with mineral waters from different thermal spring waters. These cosmetics were assayed to determine their physicochemical parameters and rheological characteristics. An accelerated oxidation test was realized to determine their oxidation stability.

## 2. Materials and Methods

### 2.1. Collection of Samples and Reagents

*Cytisus scoparius* flowers and *Quercus robur* L. acorns without cupules were manually collected in Outeiro de Rei (Lugo, Spain), whereas *Pleurotus ostreatus* mushrooms and *Brassica rapa* var. *rapa* leaves were supplied for a local grocery store (Ourense, Spain). All samples were cold stored (−18 ± 2 °C) until further use, apart from *Q. robur* which was maintained at room temperature. Prior to the MHG process, frozen samples were defrosted at 4 ± 2 °C for 24 h whereas acorns were manually chopped with a longitudinal incision and divided into quarters. In all cases, the employed reagents were of analytical grade.

### 2.2. Extraction Procedures

Microwave hydrodiffusion and gravity was used to extract the bioactive fractions of tested raw materials. For this purpose, the extractions were made on a multimode microwave extractor (NEOS-GR, Milestone Srl, Italy) as reported elsewhere [22]. Samples (100 g) were placed on an open vessel (1.5 L) and submitted at a power density of 1 W/g. These conditions were selected based on the results previously reported for other similar materials [23]. The collected volume (each 5 mL), time and temperature were recorded in all cases. Note that the MHG process was ended whenever it was not possible to collect more liquid extract. Liquors were drained by gravity on a condenser outside the microwave. Extracts were cold-stored at 4 ± 2 °C in the absence of light until further analysis. Experiments were made at least in duplicate.

An approximation of the extraction procedure’s environmental influences [24] and energy requirements [25] were calculated as previously reported. In the first case, the required dioxide carbon released was determined assuming 800 g of CO_2_ by the consumed of 1 kWh from coal or fuel. Concerning energy consumption, it was calculated as the necessary time by the device power necessary for each trial.

For comparative purposes, samples (1 g) were submitted to solid-liquid extraction (SLE) with ethanol solution (10 mL) (Sigma-Aldrich Corp., St. Louis, MO, USA). Note here that *B. rapa* leaves were treated with 90% (*v*/*v*) ethanol solution whereas the remaining samples were extracted with 70% (*v*/*v*) ethanol solution in accordance with previous assays. This solvent extraction was performed at 150 rpm and 40 ± 2 °C for 24 h in an orbital incubator shaker (Innova 4000, New Brunswick Scientific, Edison, NJ, USA) in the absence of light conditions. Collected extracts were filtered and kept at 4 ± 2 °C in darkness until further analysis.

### 2.3. Extracts Characterization

The procedures used to characterize the collected liquid extracts extracted by MHG and by SLE procedures are presented in the following sections.

#### 2.3.1. pH

pH values were determined using a pH meter (GLP 21, Hach Lange Spain, S.L.U., Barcelona, Spain) at room temperature. Previously, a calibration was realized with the corresponding standard solutions.

#### 2.3.2. Total Solid Content

Total solid extraction yield (g extract/g raw material dry weight) was calculated based on the initial moisture of the raw materials utilized, the collected volume of the different extracts and the total solid content (g dry residue/L) determined gravimetrically with an extract aliquot (1 mL) by oven drying at 105 °C during 24 h.

#### 2.3.3. Bioactive Profile

The total phenolic content of the tested liquid extracts was made by the Folin–Ciocalteu test [26], expressing the obtained content as milligrams of gallic acid equivalents (GAE). For this test, samples (0.50 mL) or the standard (gallic acid) (Sigma-Aldrich Corp., St. Louis, MO, USA) was incorporated to distilled water (3.75 mL), Folin–Ciocalteu reagent (0.25 mL, 1:1, *v*/*v*) (Panreac Química, S.L.U., Castellar del Vallès, Barcelona, Spain) and sodium carbonate solution (0.50 mL, 10%, *w*/*v*) (Sigma-Aldrich Corp., St. Louis, Missouri, USA). After keeping the mixtures in the absence of light at room temperature for about 60 min, the absorbance measurements were made at 765 nm.

Concerning the antioxidant profile, the ABTS (2,2′-azino-bis(3-ethylbenzothiazoline-6-sulfonic acid) diammonium salt radical cation scavenging capacity was also determined [27], expressing the results as Trolox equivalents antioxidant capacity (TEAC). In this case, samples (20 µL) or the standard (Trolox) (Sigma-Aldrich Corp., St. Louis, MO, USA) were mixed with diluted ABTS^+^ solution (2.00 mL) (Sigma-Aldrich Corp., St. Louis, MO, USA). Mixtures were maintained at 30 ± 2 °C for 6 min before reading the absorbance at 734 nm.

The reduction of the ferric 2,4,6-tripyridyl-s-triazine (TPTZ) complex was conducted for the above samples using the ferric reducing antioxidant power (FRAP) method [28]. Note that the ascorbic acid (Merck KGaA, Darmstadt, Germany) and iron (II) sulphate heptahydrate (Sigma-Aldrich Corp., St. Louis, MO, USA) were employed as standards. Liquid extracts of 100 µL were added to 3.00 mL of the FRAP reagent. After 6 min at room temperature, the absorbance was read at 593 nm.

The DPPH (α,α-diphenyl-β-picrylhydrazyl) radical scavenging activity of the above liquors was also determined [29]. Samples (50 µL) were added to the DPPH radical solution (2 mL) (Sigma-Aldrich Corp., St. Louis, MO, USA). Afterward, the absorbance decrease was recorded at 515 nm from 0 to 16 min using a blank sample (distilled water).

The determination of the total carotenoid content of the different extracts was realized following Khosa et al. [30] method with some modifications. In few words, 1 ± 0.01 g of each sample was submitted at a liquid-liquid extraction with 50 mL of *n*-hexane (Panreac Química, S.L.U., Castellar del Vallès, Barcelona, Spain)/acetone (Carlo Erba Reagents, S.A., Sabadell, Barcelona, Spain)/absolute ethanol (Sigma-Aldrich Corp., St. Louis, Missouri, USA) (2:1:1, *v*/*v*/*v*) for 20 min at 200 rpm in darkness conditions using an orbital shaker (OL30-ME, Ovan, Barcelona, España), then was centrifuged at 4000 rpm for 10 min and measured at 420 nm. *β*-carotene (Thermo Fisher Scientific, Geel, Belgium) was utilized as standard and results were expressed as µg *β*-carotene/g raw material dry weight.

All above analytical determinations were carried out on a spectrophotometer Hitachi U-2000 (Tokyo, Japan) at least in triplicate. This point was also applicable to the next spectrophotometric methods.

#### 2.3.4. Capacity of Elastase and Tyrosinase Enzymatic Inhibition

The capacity of elastase and tyrosinase enzymatic inhibition of the studied extracted samples were conducted by an external service following the indications reported with slight modifications by Liyanaarachchi et al. [31] and Chiari et al. [32], respectively.

#### 2.3.5. Macronutrients Measurements

MHG and SLE extracts were subjected to certain analyses to determine their main components. Their monosaccharide composition was assayed by high-performance liquid chromatography (HPLC) following a modified Balboa et al. [33] method using an Agilent equipment with a differential refractive index detector. Aminex HPX-87H and Aminex HPX-87P columns (300 × 7.8 mm, Bio-Rad Laboratories, S.A., Madrid, Spain) were employed by the separation of the different carbohydrate compounds eluted with 3 mM H_2_SO_4_ (Merck KGaA, Darmstadt, Germany) at 0.6 mL/min operating at 50 ± 2 °C and deionized water at 0.4 mL/min operating at 80 ± 2 °C, respectively. Samples were neutralized with barium carbonate (Thermo Fisher Scientific, Geel, Belgium) before analysis on the Aminex HPX-87P column. Their oligosaccharide composition was determined following the Garrote et al. [34] method and consequently, samples (2.4 ± 0.10 g) were submitted to a previous hydrolysis treatment with 4% (*w*/*w*) sulphuric acid (Merck KGaA, Darmstadt, Germany) at 121 ± 2 °C during 40 min. The estimated concentrations were calculated by comparison of the spectra of D(+)-glucose anhydrous (Scharlab, S.L., Sentmenat, Barcelona, Spain), D(+)-xylose (Sigma-Aldrich Corp., St. Louis, MO, USA), D(+)-galactose (Panreac Química, S.L.U., Castellar del Vallès, Barcelona, Spain), L-rhamnose monohydrate (Sigma-Aldrich Corp., St. Louis, MO, USA), L(+)-arabinose (Sigma-Aldrich Corp., St. Louis, MO, USA), D(+)-mannose (Thermo Fisher Scientific, Geel, Belgium) and D(−)-fructose (Sigma-Aldrich Corp., St. Louis, MO, USA) commercial patterns. Data analysis was performed by Agilent ChemStation Revision B.04.03 SP1 software.

Total protein content was assessed following the procedure detailed by Bradford [35] with some changes. In short, Bradford reagent (0.4 mL) (Sigma-Aldrich Corp., St. Louis, MO, USA) was incorporated with each sample (1.6 mL). Then, the mixture was kept at room temperature for 5 min and the absorbance of this mixture was recorded at 595 nm. The same protocol was employed with bovine serum albumin (BSA) (Sigma-Aldrich Corp., St. Louis, MO, USA) as standard. The data were reported as mg BSA/ g raw material dry weight.

Total lipid content was colorimetrically determined following Kamal [36] analysis method with some modifications. Concisely, samples or standard (lauric acid) (500 µL) (Merck KGaA, Darmstadt, Germany) were vortexed with *n*-hexane (1500 µL) (Panreac Química, S.L.U., Castellar del Vallès, Barcelona, Spain) for 1 min. After, the absorbance of the mixture was measured at 204 nm. The outcomes were expressed as g lauric acid/g raw material dry weight.

#### 2.3.6. Color

The color measurements of all extracted liquid fractions were made within the CIEL*a*b* space using a colorimeter (CR-400, Konica Minolta, Japan). The obtained parameters were the lightness (whiteness, L* = 0, or brightness, L* = 100 degree), the red/green coordinate (degree of redness, a* > 0, or greenness, a* < 0) and yellow/blue coordinate (degree of yellowness, b* > 0, or blueness, b* < 0). Moreover, the corresponding magnitudes (Equations (1)–(3)) such as hue (h (°)), chroma (C*) and saturation (S*) were calculated as indicated below.

(1) $$h\left({}^{\circ}\right)=\arctan\left(\frac{b^{*}}{a^{*}}\right).$$

(2) $$C^{*}=\sqrt{\left(a^{*}+b^{*}\right).}$$

(3) $$S^{*}=\frac{C}{L^{*}}.$$

Additionally, the total color (ΔE*) and hue (ΔH*) differences (Equations (4) and (5)) were determined as follows.

(4) $${\Delta E}^{*}=\sqrt{{\left({\Delta L}^{*}\right)}^{2}+{\left({\Delta a}^{*}\right)}^{2}+{\left({\Delta b}^{*}\right)}^{2}}.$$

(5) $${\Delta H}^{*}=\sqrt{{\left({\Delta E}^{*}\right)}^{2}-{\left({\Delta L}^{*}\right)}^{2}-{\left({\Delta C}^{*}\right)}^{2}}.$$

#### 2.3.7. Sun Protection Factor

Sun protection factor (SPF) was measured following Kaur and Saraf [37] methodology. The extract aliquots (20 µL) were homogeneously blended with 40% (*v*/*v*) ethanol solution (1980 µL) (Sigma-Aldrich Corp., St. Louis, Missouri, USA) and scanned between 290 and 320 nm. According to the Mansur et al. [38] procedure, the SPF values were determined using the following equation:

(6) $$\mathrm{SPF}=\mathrm{CF}\;x\overset{320}{\underset{290}{\sum }}\mathrm{EE}\;\left(\lambda \right)\times \;I\;\left(\lambda \right)\;\times \;\mathrm{Abs}\;\left(\lambda \right).$$

where SPF corresponds to the spectrophotometric sun protection factor, CF represents a necessary correction factor (10), EE(λ) indicates the erythemal effect of the radiation with wavelength λ, I(λ) represents the solar intensity of the spectrum and Abs (λ) displays the spectrophotometric absorbance values at wavelength λ. The normalized product of EE(λ) and I(λ) utilized in the calculation of SPF was previously considered by Sayre et al. [39].

### 2.4. Formulation of the Creams

Sunscreen creams were elaborated at least in duplicate according to Balboa et al. [40] formulation. Briefly, components of oil phase (cream basis (O/W) (Derex, S.A., Rafelbunyol, Valencia, Spain) −18 g−, dimethicone 350 (Fagron Ibérica S.A.U., Terrassa, Barcelona, Spain) −6 g−, avocado oil (Fagron Ibérica S.A.U., Terrassa, Barcelona, Spain) −3 g−, sunscreen (Guinama, S.L.U., La Pobla de Vallbona, Valencia, Spain) −8 g−, micronized titanium dioxide (Fagron Ibérica S.A.U., Terrassa, Barcelona, Spain) −18 g− and fenonip XB (Fagron Ibérica S.A.U., Terrassa, Barcelona, Spain) −0.35 g−) were mixed and tempered at 70 ± 2 °C in a water bath. On the other hand, ingredients of water phase (distilled or thermal spring water −80 g−, carbomer 940 (Fagron Ibérica S.A.U., Terrassa, Barcelona, Spain) −1.5 g−, propyleneglycol (Guinama, S.L.U., La Pobla de Vallbona, Valencia, Spain) −6 g− and triethanolamine (Fagron Ibérica S.A.U., Terrassa, Barcelona, Spain) −1.5 g−) were also blended and adjusted at 40 ± 2 °C. When the oil phase mixture was completely melted then it was transferred to the water bath at 40 ± 2 °C until its temperature adjustment. In this point, this phase was added at the water phase until their homogenization promoting a gel-like matrix. The studied extracts from the different raw materials by MHG at 100 W, butylhydroxytoluene (BHT) (Roig Farma, S.A., Terrasa, Barcelona, Spain) and (±)-*α*-tocopherol (Sigma-Aldrich Corp., St. Louis, MO, USA) commercial antioxidants or the different used water as controls (750 µL or mass equivalent weight), bergamot oil (450 µL) (Fagron Ibérica S.A.U., Terrassa, Barcelona, Spain) and tetramer cyclomethicone (3 mL) (Fagron Ibérica S.A.U., Terrassa, Barcelona, Spain) were mixed in the O/W emulsion at room temperature and thoroughly mixed. The final product was packed in flasks and amber glass vials and stored at refrigeration temperature until their analysis.

#### Water Features

Thermal spring waters were collected in plastic bottles in their points of emission (three different points of the Ourense region) and kept at 4 ± 2 °C in the laboratory within less the next 1 h after their gathering.

The electrical conductivity values of waters were determined by a conductivity meter (HI 9033, Hanna Instruments Ltd., Eden Way, UK) at room temperature. pH determination, total solid content level and color features of the different used waters were measured as indicated above.

### 2.5. Creams Characterization

Freshly elaborated sunscreen creams were examined so that their pH value, total solid content, SPF data, color characteristics, thiobarbituric acid reactive substances (TBARS) and viscous properties were determined. The effect of the elevated temperature and storage time on the stability of these samples was determined by means of accelerated oxidation at 50 ± 2 °C for 15 days. The pH and TBARS kinetics tests were done periodically on 1-day intervals whereas total solid content and color features assays were defined at the end of this process.

pH and color tests of the emulsions were realized as indicated above. The total solid content was determined using a portion of cream (0.5 g) under the above conditions. Sunscreen cream SPF values were also analyzed using a part of sunscreen cream (1 g) and 96% (*v*/*v*) ethanol (Sigma-Aldrich Corp., St. Louis, MO, USA) up to 25 mL following Dutra et al. [41] indications. The solution was stirred at 500 rpm for 5 min, treated by ultrasounds for 5 min and filtered. Absorbance data were read between 290 and 320 nm at 5 nm intervals. In this case, the correction factor was 20 and consequently, this value was considered in the mathematical calculation (Equation (6)). This value corresponded to a standard sunscreen formulation with a 5.46% solar filter equivalent to an SPF value of 3.18.

In order to evaluate the effectiveness of selected MHG extracts against lipid oxidation, thiobarbituric acid reactive substances (TBARS) was utilized following the assay of Scheffler et al. [42]. Shortly, samples or standard (malonaldehyde solution (20 nmol/mL) (Sigma-Aldrich Corp., St. Louis, MO, USA)) (0.8 g) were mixed with thiobarbituric acid-butylated hydroxytoluene (TBA-BHT) solution (1.6 mL) in tubes and shaken for 30 s using a vortex. This solution was prepared with 4,6-dihydroxy-2-mercaptopyrimide (15 g) (Thermo Fisher Scientific, Geel, Belgium), hydrochloric acid 12 M (1.76 mL) (Thermo Fisher Scientific, Geel, Belgium), distilled water (82.9 mL) and BHT (2%, *v*/*v*) (3 mL) (Roig Farma, S.A., Terrasa, Barcelona, Spain), previously treated with absolute ethanol (Sigma-Aldrich Corp., St. Louis, MO, USA). Afterward, test tubes were submitted to 95 ± 2 °C for 15 min in a water bath, cooled in an ice-tap-water bath for 10 min and centrifuged at 5000 rpm for 10 min. The absorbance of the supernatant was measured at 532 nm. The concentration of TBARS was expressed as nmol malonaldehyde/g sun cream dry weight.

The viscous profile of all formulated creams was determined. For this purpose, the apparent viscosity vs. shear rate (‘so-called’ steady-shear flow curves) was monitored at 25 °C on a controlled-stress rheometer (MCR 302, Paar Physica, Austria). The temperature was controlled by a Peltier system (± 0.01 °C). The selected measuring system was a sandblasted plate-plate (25 mm diameter, 1 mm gap) to avoid a possible slip of the samples. The edges of all creams placed on the plate-plate geometry were sealed with light paraffin oil to avoid water loss during experiments and were rested for 10 min to allow sample temperature and structural equilibration. In order to study the hysteresis effects, all steady-state shear measurements were made by decreasing and, subsequently, increasing shear rate following a logarithm. All viscous experiments were performed at least in triplicate.

### 2.6. Statistical Analysis

Experimental data were studied using one-factor analysis of variance, ANOVA (PASW Statistics v.22, IBM SPSS Statistics, New York, NY, USA). If the variance study indicated means differences, a Scheffé test was made to distinguish means with 95% confidence (*p* < 0.05).

## 3. Results and Discussion

### 3.1. Microwave Hydrodiffusion and Gravity (MHG)

**Figure 1:**
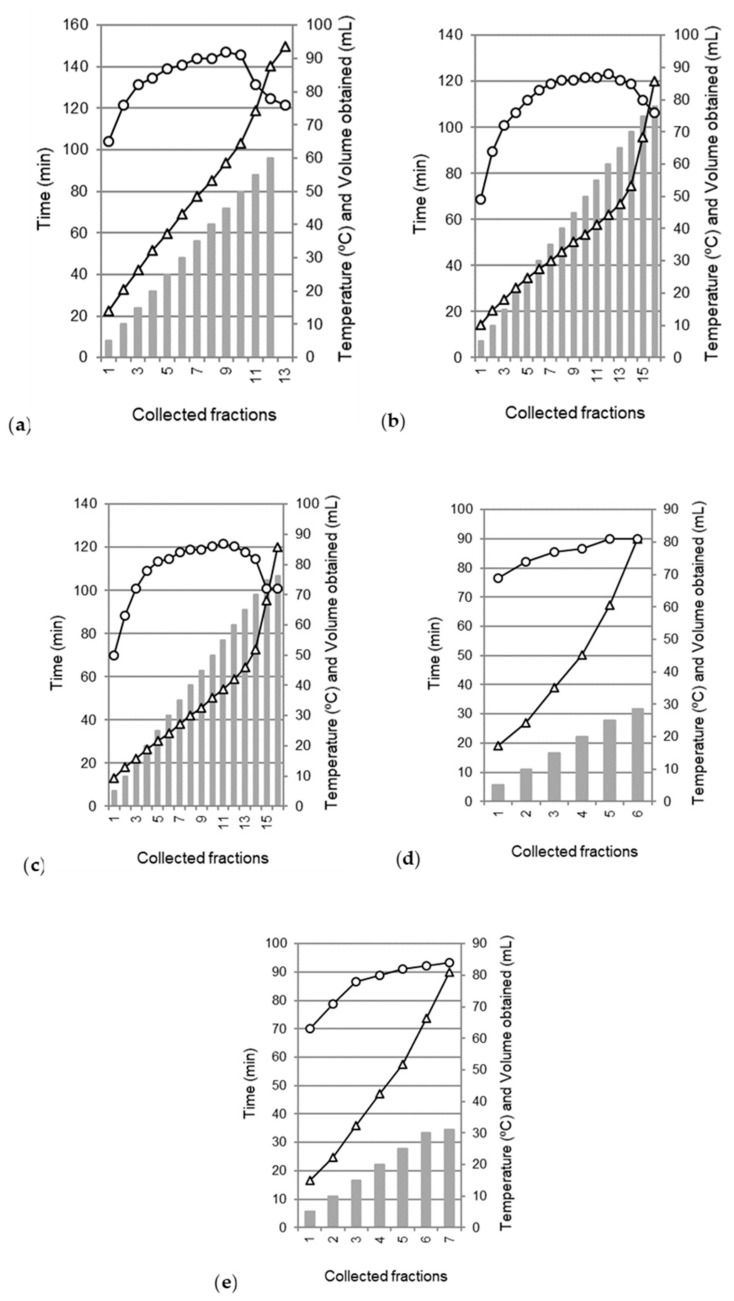
Collected volume (bars), vessel temperature (circles) and extraction time (triangles) during MHG treatment at 100 W of irradiation power of different raw materials: (**a**) defrosted *Cytisus scoparius* flowers, (**b**) defrosted fruiting bodies of *Pleurotus ostreatus* mushrooms, (**c**) defrosted *Brassica rapa* L. var. *rapa* leaves, (**d**) whole and (**e**) divided into quarters *Quercus robur* L. acorns. Note here that y-axes scales are different in each plot.

Extraction time and temperature values by MHG from different samples are showed in Figure 1 in combination with the data of gathered extract volume. *Cytisus scoparius* flowers (Figure 1a) treated at 100 W provided 62.2 mL of total liquid phase for 150 min reaching a maximum temperature data of 92 °C. This raw material presented the typical microwave heating profile identified for other sources. Firstly, a latency period was passed after the first drop of liquid phase outside the microwave oven (around 8 min at 33 °C with a heating rate of 1.56 °C/min). Secondly, the next phase could be collected about 32% of the total volume for approximately 43 min. The following step exhibited a plateau region characterized by an average of 90 °C during ~52 min (heating rate of 0.14 °C/min) obtaining 48% of the total extract. At the last interval, the temperature decreased with a heating rate of 0.32 °C/min. This step (47 min) meant close to 20% of the total aqueous extract. Similar behavior was also displayed for *Pleurotus ostreatus* mushrooms and *Brassica rapa* leaves (Figure 1b,c). These samples, submitted to an MHG extraction process of 120 min, supplied 78 and 76.3 mL, respectively. The heating outline derived by the whole and divided into quarter acorns of *Quercus robur* L. did not describe the last phase since their moisture content (32.27 ± 3.79% wet basis, w.b.) was much lower than the other matrices. This variable is disclosed as a decisive element since in situ water of the tissue cells permits isolate the natural valuable compounds behaving like their transport media [43]. Processed acorns during 90 min exhibited roughly 30 mL of the liquid phase. The size reduction pretreatment of the samples favored slightly (9%) the extraction of their extracts since the influence of particle size is a key factor on the optimization of the extraction process as reported in the extraction of antioxidant compounds of other material such as tea or ginger by solid-liquid extraction [44].

The impact of MHG at 100 W on the liquid phase collected is displayed in Figure S1a. Samples provided a range of the initial volume of achieved water of 75–97%. The difference between the results was related to their moisture content. Note here that the moisture content of *C. scoparius* flowers, *P. ostreatus* mushrooms, and leaves of *B. rapa* was 82.79% ± 0.29% w.b., 89.43% ± 0.23% w.b. and 90.70% ± 0.71% w.b., respectively. These data agreed with those reported for other materials as the brown seaweed *Undaria pinnatifida* under similar extraction conditions [45]. The maximum total solid extraction yield (about 5.1 mg extract/g raw material dry weight) was obtained when mushroom samples were used (Figure S1b). Their structure tissue favored the removal of bioactive compounds in comparison with the other matrices. For example, their yield was ~5 times higher than the value derived by acorns samples. This pattern was also attributed to the energy consumption for the processing of these types of raw materials (Figure S1c) since acorns needed the lowest energy requirements (roughly 540 kJ). The energy consumption was elevated to approximately 900 kJ for to treat the wildflowers. The environmental impact defined as the quantity of carbon dioxide rejected into the atmosphere was in order to 120–200 g. This reflected the reduced burden of MHG extraction technology compared to conventional extraction methods [46].

### 3.2. Antioxidant Properties of the Gathered Liquid Extracts

**Figure 2:**
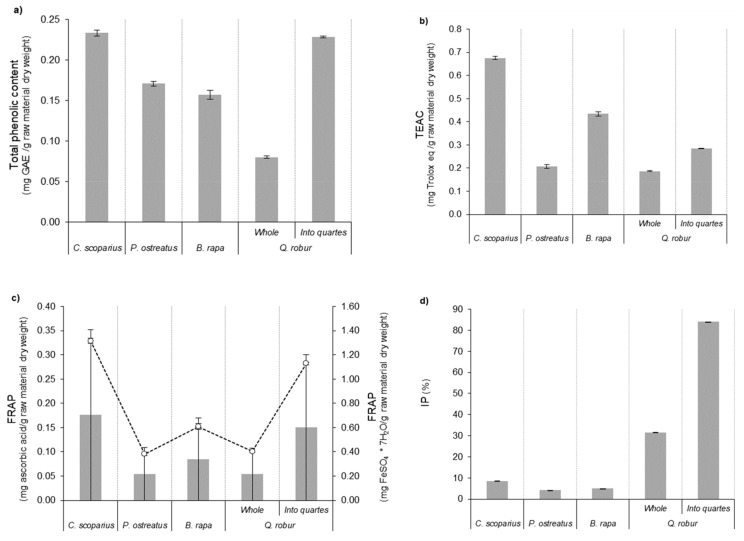
Total phenolic content (**a**) and antioxidant capacity, expressed as TEAC value (**b**), FRAP assay (**c**) as ascorbic acid equivalents (circles) and as ferric sulphate equivalents (bars) and inhibition percentage (IP) of DPPH radical (**d**), from the aqueous collected of the different took in consideration raw materials at 100 W by MHG extraction.

The total phenolic content and antioxidant potential of the extracts from the raw materials treated at 100 W by MHG technology are collected in Figure 2. Extracts from *C. scoparius* flowers showed the maximum total phenolic content, TEAC and FRAP values (around 0.23 mg GAE/g raw material dry weight, 0.68 mg Trolox eq/g raw material dry weight and 0.33 mg ascorbic acid/g raw material dry weight or 0.71 mg FeSO_4_ × 7H_2_O/g raw material dry weight, respectively). Their total phenolic content agrees with the data previously found [12] for hydromethanolic and ethyl lactate extracts obtained by pressurized liquid extraction. On the other hand, *P. ostreatus* and *B. rapa* displayed similar results when both are compared. Specifically, their ferric reducing antioxidant power outcomes did not present significant differences among them (their average was about 0.12 mg ascorbic acid/g raw material dry weight or 0.28 mg FeSO_4_ × 7H_2_O/g raw material dry weight). *B. rapa* showed the greatest antioxidant capacity with a noticeable concentration of 0.43 mg Trolox eq/g raw material dry weight and a total phenolic content higher than those reported by this sample [47]. Outcomes from the acorns of *Q. robur* indicated that samples divided into quarters provided a total phenolic content like the maximum data of wildflowers. This value was higher than reported by Rakić et al. [48] for similar raw materials submitted to methanolic extraction. This type of sample kept this tendency in the antioxidant capacity determined by the different free radical scavenging assays. The difference between the whole and the divided acorns was around 0.1 mg Trolox eq/g raw material dry weight for TEAC determination and 0.18 g ascorbic acid/g raw material dry weight or 0.39 mg FeSO_4_ × 7H_2_O/g raw material dry weight for FRAP test. The DPPH percentage inhibition variation of the collected volume extracts from the two types of acorns was close to 57.8%. In this case, the divided raw material gave an effective concentration (EC_50_, defined as the concentration in which 50% of DPPH radical was scavenged) of 0.05 g dry weight/100 g sample (equivalent to 0.67 mM of ascorbic acid).

**Figure 3:**
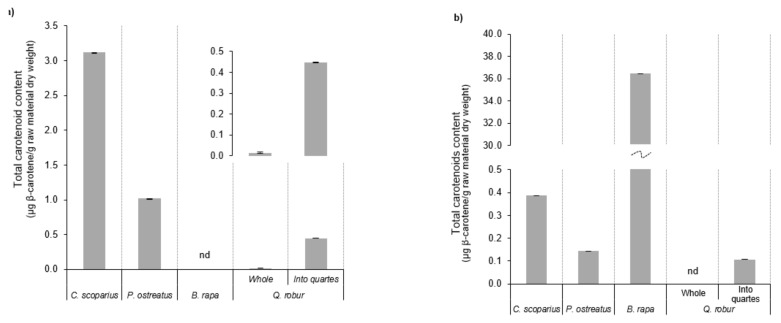
Total carotenoids content from the collected aqueous extracts of the different took in consideration raw materials at 100 W by MHG extraction (**a**) and ethanol extracts collected by SLE (**b**) of the evaluated feedstocks. nd: no detected.

Carotenoids are pigments integrated by lipid-soluble tetraterpenoid compounds. Their structure can affect their antioxidant potential scavenging efficiently O_2_ and peroxyl radicals [49,50]. The total carotenoid content of the liquid extracts is summarized in Figure 3a. Wildflowers provided the maximum concentration (3.1 µg *β*-carotene/g raw material dry weight). This value could be attributed to its antioxidant profile since it was more elevated as stated above. In comparison with mushroom data, their concentration was around three times superior-this relation differed to their antioxidant profile-. The samples of acorn presented the lowest total carotenoid content: the samples divided in quarters allowed a result of about 32.3% higher than the whole nuts. In the current study, the raw materials used by MHG procedure were also submitted to a solid-liquid extraction (SLE) with 70–90% (*v*/*v*) ethanol solution as a solvent for evaluation purposes. The resultant total carotenoid content appears in Figure 3b. In contrast with the MHG outcomes, *B. rapa* samples supplied the maximum concentration registered of total carotenoids content (approximately 3.1 µg *β*-carotene/g raw material dry weight). This value was the highest outcome described by *Brassica rapa* ssp. *chinensis* sprouts [51]. The opposite trend was followed by the remaining samples since these showed issues lower than the reported by MHG procedure (with a difference in the range between 0.34 and 2.73 units).

**Figure 4:**
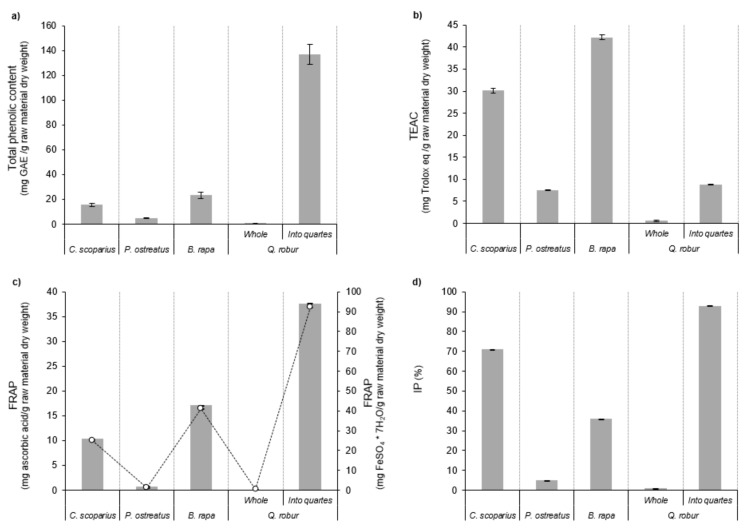
Total phenolic content (**a**) and antioxidant capacity, expressed as TEAC value (**b**), FRAP assay (**c**) as ascorbic acid equivalents (circles) and as ferric sulphate equivalents (bars) and inhibition percentage (IP) of DPPH radical (**d**), from the ethanol extracts by SLE of the different raw materials.

The antioxidant profile of the ethanolic extracts from the different employed resources by SLE is exhibited in Figure 4. Despite the fact that the total phenolic content of the *C. scoparius* flowers was limited (close to 16 mg GAE/g raw material dry weight), their antioxidant potential was defined by considerable TEAC and DPPH values (around 30 mg Trolox eq/g raw material dry weight and 71.0% inhibition percentage, respectively). Their EC_50_ was 0.95 ± 0.01 g/100 g dry weight (equivalent to 0.60 mM of ascorbic acid). Note here that these wildflowers showed the maximum total solid extraction yield (close to 5.8 mg extract/g flower dry weight). The antioxidant data from *P. ostreatus* samples did not stand out when compared with the other raw materials, although their concentrations were higher than the values obtained by MHG. This agrees with the tendency described by the other sources. On the other hand, *B. rapa* allowed the largest TEAC parameter (42.2 mg Trolox eq/g raw material dry weight) whereas their FRAP and DPPH magnitudes were intermediate (16.5 mg ascorbic acid/g raw material dry weight or 42.8 mg FeSO_4_ × 7H_2_O/g raw material dry weight and 36.0% inhibition percentage, respectively). Even though, *Q. robur* acorns divided into quarters had not an important TEAC concentration, this sample provided the maximum data of total phenolic content (136.9 mg GAE/g raw material dry weight), FRAP values (36.9 mg ascorbic acid/g raw material dry weight or 94.1 mg FeSO_4_ × 7H_2_O/g raw material dry weight) and DPPH inhibition percentage (93.0%) with significant difference in comparison with the remaining resources. Overall, it is necessary to emphasize that a strong positive correlation was displayed between total phenolic content and ascorbic acid and iron (II) sulphate heptahydrate results (r = 0.950 and r = 0.948, respectively).

### 3.3. Proximate Chemical Profile

**Figure 5:**
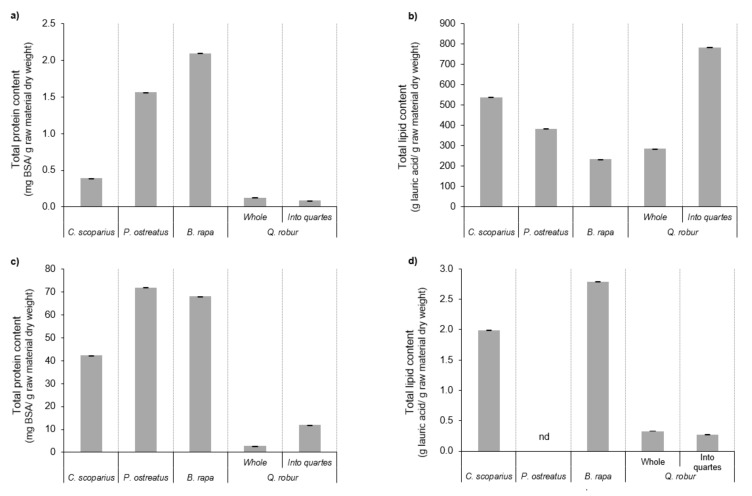
Total protein content (**a**,**c**) and total lipid content (**b**,**d**) from the collected aqueous extracts of the different took in consideration raw materials at 100 W by MHG extraction and from the SLE ethanolic extracts, respectively, from different raw materials. nd: no detected.

The collected aqueous extracts by the MHG method and ethanol extracts derived by SLE extraction were submitted to macronutrients analysis. Their total protein and total lipid contents are presented in Figure 5. *B. rapa* and *P. ostreatus* samples showed the highest total protein content by MHG and SLE extraction procedures, respectively. These data varied between approximately 2.1 and 72.0 mg BSA/g raw material dry weight. Generally, the tendency of SLE extracts corresponded with the behavior defined by their total carotenoid content. This trend could be defined by the chemical structure of the vegetable pigments since they are linked to some chlorophyll-carotenoid proteins [47]. In relation to total lipid content of the gathered extracts, *Q. robur* divided hard fruits supplied the highest outcome by MHG (783.9 g lauric acid/g raw material dry weight) with significant differences as compared with the other feedstocks. The ethanolic extracts provided the lowest total lipid content issues. The obtained data showed that ethanol solvent at 40 ± 2 °C did not permit recovering lipid compounds from the different samples. These reduced lipid yields could be improved using a multiple-batch sequence of organic solvents at greatest temperature ranges able to removal apolar compounds as occurred with other raw materials as rice bran or coffee grounds [52,53]. Further studies should be interesting to increase the lipid extraction from the used raw materials.

The monosaccharide and oligosaccharide composition of the extracts from MHG and SLE methodologies are summarized in Table 1. The sample that presented the highest carbohydrate concentration was *B. rapa* followed by *P. ostreatus* and *C. scoparius*. Overall, SLE could extract higher quantities of carbohydrate compounds than the MHG procedure. Mannose was the main monosaccharide identified in all samples. In all cases, the corresponding oligosaccharide was also the major compound. Turnip sample displayed a higher concentration of oligosaccharides than monosaccharides (about double times). This behavior was in accordance with the literature data from *Brassica oleracea* leaves [54]. Different studies described that mannose is present in the structure of the functional protein as ligands or signaling molecules [55,56]. A similar tendency was observed in this monosaccharide and the total protein content of SLE extracts suggested a relation between both components. The extraction capacity of the MHG method varied between around 0.02% and 95.65% as well as between 0.05% and 0.44% of the total recovery monosaccharides and O-mannose compounds obtained by SLE. *Q. robur* acorn exhibited the lowest data. It is worth noting that MHG whole acorns aqueous extracts displayed an opposite trend in their xylose, rhamnose and mannose monosaccharides concentration. This sample divided in quarters also shared this tendency for the values of their rhamnose and O-glucose oligosaccharide compounds.

### 3.4. Color Characteristics of the Extracts

Colorimetric coordinates from the CIEL*a*b* method were measured directly to the extracts from the different samples by MHG at 100 W and SLE extractions. Considering the above indices, the color magnitudes were calculated according to the equations stated above. These data are reported in Table 2. In general, lightness, a* coordinates and the hue angle of the ethanolic extracts by SLE showed lower values than the aqueous extracts by MHG. The opposite tendency was observed by the b* coordinate and Chroma and saturation parameters. Significant differences were identified between the tested extraction procedures for each resource. Particularly, the hue angle of ethanolic extract from acorns divided into quarters disclosed higher differences between the two extraction systems (64.9°). The variation of the color features of the gathered extracts was also clear when the total color difference (ΔE*) of the samples from MHG was compared with the parameters derived by the SLE technique, according to the classification of Adekunte et al. [57]. This change was classified as a small difference (ΔE* < 1.5) for whole acorns and different (1.5 < ΔE* < 3.0) for divided nuts and mushrooms. These results could indicate that these extraction methodologies provided similar color extracts from these samples. However, the application of MHG and SLE processes from wildflowers and turnip leaves could practice an effect more dissimilar under these plant matrices since their value of total color difference was very different (ΔE* > 3.0). Concerning the hue difference (ΔH*) carried out with a trend like the total color difference in order that the *Q. robur* acorns and *B. rapa* samples presented the lowest and the highest hue difference values, respectively. It is necessary to note that a strong negative correlation between L* and a* coordinates and total carotenoid content was noticeable (r = −0.912 and r = −0.982, respectively). Conversely, a positive correlation with this concentration and the b* coordinate, C* and S* magnitudes was identified (r = 0.971–0.987). Based on these Pearson’s correlation coefficients, the relationship of total carotenoid content with parameters from the CIELab color space will allow estimate rapidly this pigment concentration of extracts by MHG and SLE technologies with a non-destructive method. Similar associations were established for several vegetable raw materials [58,59].

### 3.5. Utilization of MHG Extracts on Cosmetic Formulations

Natural extracts obtained from underused and by-products raw materials can act as functional additives in cosmetic products contributing to healthy properties. These sustainable sources are not expensive and appear in abundance. The environmental, social and economic impact of the production of these extracts is also evaluated by cosmetic industries since nowadays the preservation of the environment and a positive influence on society sphere are objectives to be achieved. Currently, there is a growing interest in the incorporation of these products in cosmetic formulations in response to the increasing demand by consumers [40,60]. The potential cosmeceutical use of the *C. scoparius*, *P. ostreatus*, *B. rapa* and *Q. robur* acorns divided into quarters MHG extracts was researched by the incorporation of this aqueous extracts as an additive ingredient at sunscreen cream formulations. The added extracts displayed an approximately pH value on the range from 3.1 to 5.3. Their SPF data varied between approximately 0.04 and 0.21. It is remarkable that flower extract provided the highest SPF issue whereas their pH was the lowest. Note here that the SPF value of the elaborated creams was around 5.8. Concerning the enzymatic inhibition assays (data not shown), the *Q. robur* sample provided the highest anti-elastase capacity with an effective concentration (EC_50_) of 606 mg extract/L (equivalent to 2.3 mg epigallocatechin gallate/L) and an EC_50_ tyrosinase inhibitory capacity of 496 mg extract/L (equivalent to 93 mg kojic acid/L). These preliminary results suggested that this extract may be helpful in preventing the loss of skin sagging and elasticity and the pigmentation damage [61].

These O/W emulsions were formulated with three different types of thermal spring waters and with distilled water for comparative purposes. Their pH values ranged from 5.46 to 7.57. These parameters were in accordance with the values of the pH reported by Delgado-Outeiriño et al. [62] for different samples from thermal spring waters of the same region. The electrical conductivity of the thermal water samples varied on the range of 1260–3200 µS so that the thermal spring water 1 displayed the highest value in accordance with their total solid content (0.45 mg dry residue/mL). The remaining samples exhibited an average of ~0.13 mg dry residue/mL. No significant differences were provided between these samples (data not shown). This behavior was also provided in the color magnitudes of the water samples. Their hue angle was about 136.4° whereas their Chroma and saturation were thereabouts 5.6 and 0.06, respectively.

Figure S2 discloses the total solid content of the sunscreen creams freshly processed. Results obtained showed that no significant differences were detected between the different types of water and the different extracts or commercial antioxidants. This parameter varied in a limited interval of approximately 0.31 and 0.35 g dry residue/g sun cream, respectively. These creams submitted at the accelerated oxidation at 50 ± 2 °C for 15 days displayed a total solid content lower, with minimum and maximum values of 0.328 and 0.330 g dry residue/g sun cream, respectively. In relation to the color features of the creams made at the initial time (data not shown), the measurements exhibited similar issues with small differences (ΔE* < 1.5) for their total color parameter. The highest data was provided for *P. ostreatus* and/with thermal spring water 2 cream (ΔE* = 0.84). This sample also exhibited the highest value of this same parameter with an outcome of ΔE* = 2.00 after 15 days of stability testing. In this case, the oxidation chemical reactions that were produced derived into an increase of the total color difference of the tested cosmetics, since different differences (1.5 < ΔE* < 3.0) were registered in thermal spring water 2 cream with all added antioxidant ingredients and *Q. robur* sun cream with thermal spring water 3.

Kinetics of pH values for 15 days of the accelerated assays were realized and representative values were gathered in Figure S3. Overall, the pH of the creams varied between 5.3 and 6.3. Distilled water was the type of water that offered the pH data with more fluctuation (ranged from 5.3 to 5.9) in contraposition with water from thermal spring water 3 (with a variation of about 0.2 points). These data were compatible with the normal skin pH [63]. Thermal water spring 1 control cream supplied data slightly higher than added extracts and antioxidant creams made with this same type of water. This behavior could be associated with the acid pH of the added extracts and the BHT and (±)-*α*-tocopherol features in combination with characteristics of the different used ingredients in this model cosmetic. This observation was not reflected in the evolution of the TBARS values of these same samples (Figure S4). In this case, no significant differences were recorded for the different studied emulsions elaborated with each type of water. Generally, a plateau region was described by the malonaldehyde values of the different sun creams. The data of this marker of lipid peroxidation process ranged from 11.8 to 26.7 nmol malonaldehyde/g sun cream dry weight. Several scientific studies reported a similar trend for the TBARS index in O/W emulsions [64,65].

**Figure 6:**
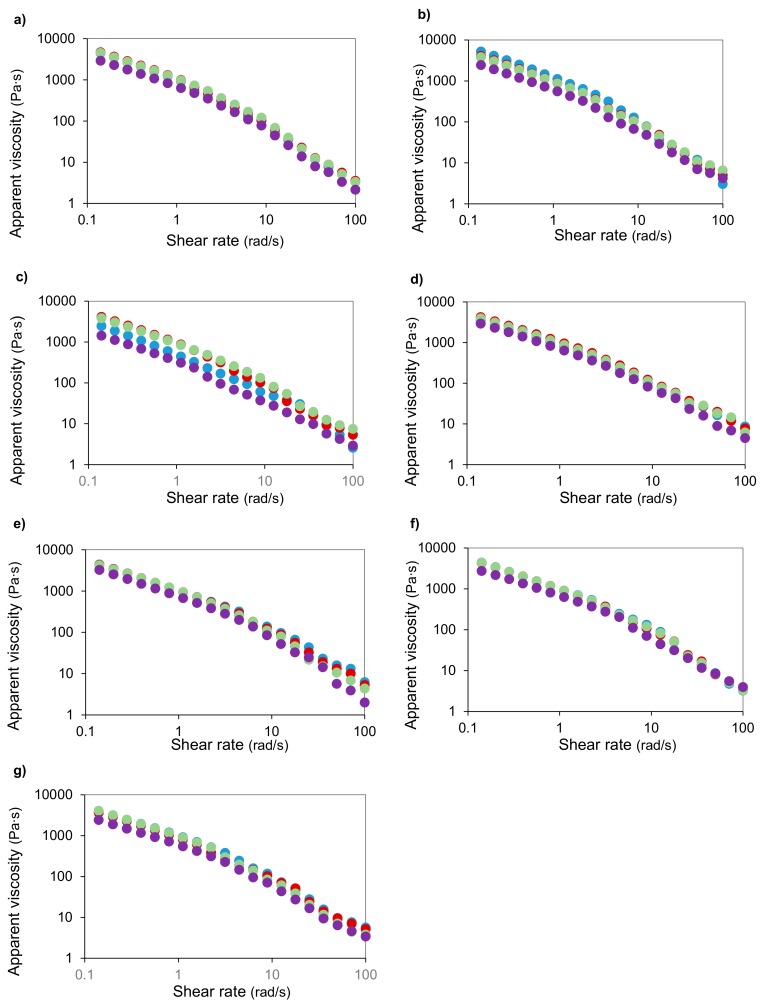
Viscous behavior of the sun cream formulated with selected MHG extracts from (**a**) *C. scoparius* flowers, (**b**) *P. ostreatus* mushrooms, (**c**) *B. rapa* leaves and (**d**) *Q. robur* acorns and (**e**) BHT, (**f**) (±)-*α*-tocopherol and (**g**) water control. Color code: distilled water (blue), thermal spring water 1 (red), thermal spring water 2 (green) and thermal spring water 3 (purple).

Figure 6 summarizes the effect of the three tested thermal waters and distilled water in the flow behavior at 25 °C of creams incorporated with different natural extracts (*C. scoparius*, *P. ostreatus*, *B. rapa*, *Q. robur*). For all tested systems, the apparent viscosity showed shear-thinning behavior, dropping around four decades with an increasing shear rate. At the fixed shear rate, the presence of thermal spring water 3 promoted the decrease of the apparent viscosity, especially for creams formulated with *P. ostreatus* and *B. rapa* (Figure 6b,c). This is an indicator of the relevance of studying different extracts because the rheological properties greatly depend on the analyzed extract and its specifically composition. This suggests that water from the thermal spring 3 can provide sunscreen creams that will be easily applicable in the skin [66,67]. Concerning magnitudes of the apparent viscosity of the creams formulated with different extracts, results suggest that those that presented higher bioactive compounds also exhibited lower apparent viscosity values. This agrees with the achievements provided for other different functional matrices [68,69,70]. Concerning BHT and (±)-*α*-tocopherol creams (Figure 6e,f), similar profiles and magnitudes were identified, whereas those made in the water control (Figure 6g) displayed slightly higher values. Again, no hysteresis loops were observed in tested sunscreen creams.

## 4. Conclusions

Emerging MHG technology was found to be appropriate to produce extracts with antioxidant properties. The highest total solid extraction yield was obtained from *P. ostreatus* mushroom whereas the lowest energy requirements were identified for *Q. robur* acorns. This last sample divided into quarters also displayed the highest DPPH data. *C. scoparius* flowers provided the extracts with the highest total phenolic content, TEAC, and FRAP values and total carotenoid content. *B. rapa* extracts from MHG was not relevant in contraposition with their ethanolic extracts that disclosed relevant bioactive properties in combination with acorns divided into quarters. These samples showed the most noticeable total lipid content by SLE and MHG, respectively. *B. rapa* supplied the highest total protein content and carbohydrate concentration by MHG extraction systems. The color features and total carotenoid content of the studied extracts presented a strong correlation. In relation to the cosmetic application of the MHG extracts, *Q. robur* acorns divided into quarters registered the highest anti-elastase and anti-tyrosinase capacities. The use of these extracts into sunscreen creams with three different thermal spring waters disclosed that their chemical and bioactive features were like those with added commercial antioxidants. The combination of *P. ostreatus* and *B. rapa* extracts with thermal spring water 3 offered the most suitable mechanical properties favoring the skin cream application. Further studies need to focus on improving the rheological properties at different temperatures over time in combination with the sensorial analysis of the formulated cosmetic emulsions. Finally, MHG lab-scale studies should be applied to pilot scale, following the suitable results previously found for the MHG technique applied to pilot-scale solvent-free microwave extraction of polyphenols from *Lettuce sativa* [71].

## Figures and Tables

**Table 1:** Influence of the extraction at 100 W by MHG and solid liquid extraction from the different raw materials on the monosaccharide and oligosaccharide composition (mg/g raw material dry weight) of the liquid phase collected.

Composition (mg/g Raw Material Dry Weight)		Extraction Technique	Raw Materials				
			*C. scoparius*	*P. ostreatus*	*B. rapa*	*Q. robur*	
						Whole	Into Quarters
**Monosaccharides**	Glucose	MHG	0.34 ± 0.00 ^j^	0.68 ± 0.00 ^i^	0.32 ± 0.00 ^m^	0.01 ± 0.00 ^g^	0.01 ± 0.00 ^k^
		SLE	10.60 ± 0.00 ^e^	2.89 ± 0.00 ^g^	9.31 ± 0.01 ^f^	0.01 ± 0.00 ^g^	0.10 ± 0.00 ^g^
	Xylose	MHG	0.08 ± 0.01 ^l^	0.17 ± 0.00 ^l^	0.17 ± 0.00 ^n^	0.03 ± 0.00 ^e^	0.01 ± 0.00 ^l^
		SLE	2.36 ± 0.01 ^h^	0.47 ± 0.01 ^j^	1.03 ± 0.01 ^i^	0.03 ± 0.01 ^e^	0.03 ± 0.00 ^i^
	Galactose	MHG	0.04 ± 0.00 ^ll^	0.07 ± 0.00 ^m^	0.12 ± 0.01 ^ñ^	0.02 ± 0.01 ^f^	0.01 ± 0.00 ^k^
		SLE	0.37 ± 0.00 ^j^	0.34 ± 0.00 ^k^	0.62 ± 0.01 ^k^	0.02 ± 0.01 ^f^	0.03 ± 0.00 ^j^
	Rhamnose	MHG	0.01 ± 0.00 ^m^	0.13 ± 0.00 ^ll^	0.05 ± 0.00 ^p^	0.01 ± 0.01 ^g^	0.03 ± 0.00 ^i^
		SLE	0.13 ± 0.00 ^k^	0.16 ± 0.01 ^l^	0.39 ± 0.01 ^ll^	0.01 ± 0.00 ^g^	0.01 ± 0.00 ^k^
	Arabinose	MHG	0.45 ± 0.00 ^i^	nd	0.44 ± 0.00 ^l^	nd	0.07 ± 0.00 ^h^
		SLE	nd	nd	nd	nd	nd
	Mannose	MHG	0.37 ± 0.00 ^j^	6.11 ± 0.03 ^f^	0.85 ± 0.00 ^j^	97.03 ± 2.78 ^b^	0.78 ± 0.00 ^e^
		SLE	1537.0 ± 2.4 ^b^	1551.3 ± 2.8 ^b^	2253.5 ± 0.8 ^b^	96.44 ± 0.80 ^b^	107.20 ± 0.37 ^b^
	Fructose	MHG	nd	1.28 ± 0.01 ^h^	0.61 ± 0.01 ^k^	0.47 ± 0.31 ^d^	0.03 ± 0.00 ^j^
		SLE	5.14 ± 0.48 ^f^	10.21 ± 0.39 ^e^	8.32 ± 0.01 ^g^	0.76 ± 0.65 ^c^	0.41 ± 0.02 ^f^
**Oligosaccharides**	O-glucose	MHG	nd	nd	0.07 ± 0.00 ^o^	0.01 ± 0.00 ^h^	0.03 ± 0.00 ^j^
		SLE	nd	0.72 ± 0.00 ^i^	nd	nd	0.02 ± 0.05 ^k^
	O-xylose	MHG	nd	nd	nd	nd	nd
		SLE	43.96 ± 0.15 ^d^	13.47 ± 0.02 ^d^	18.17 ± 0.10 ^e^	nd	0.43 ± 0.17 ^f^
	O-galactose	MHG	nd	nd	nd	nd	nd
		SLE	nd	nd	nd	nd	nd
	O-rhamnose	MHG	54.33 ± 0.31 ^c^	572.3 ± 0.0 ^c^	599.2 ± 4.4 ^d^	nd	7.16 ± 2.24 ^c^
		SLE	nd	nd	nd	nd	nd
	O-arabinose	MHG	3.42 ± 0.05 ^g^	nd	3.62 ± 0.05 ^h^	nd	0.01 ± 0.05 ^k,l^
		SLE	nd	nd	nd	nd	nd
	O-mannose	MHG	nd	2.10 ± 0.39 ^g^	nd	nd	1.19 ± 0.22 ^d^
		SLE	2880.5 ± 0.7 ^a^	4492.9 ± 0.6 ^a^	4059.0 ± 8.2 ^a^	166.5 ± 2.3 ^a^	271.2 ± 58.3 ^a^
	O-fructose	MHG	nd	nd	706.03 ± 1.62 ^c^	nd	nd
		SLE	nd	nd	nd	nd	nd

Data are given as mean ± standard deviation. Data values in a column with different superscript letters are statically different (*p* ≤ 0.05). nd: no detected.

**Table 2:** Colorimetric features by CIEL*a*b* system from the aqueous phase collected at 100 W by MHG extraction and solid liquid extraction of the different raw materials.

Raw Materials		Extraction Technique	Coordinates				Magnitudes			
			Lightness (L*)	a*		b*	Hue Angle (h°)	Chroma (C*)		Saturation (S*)
*C. scoparius*		MHG	90.10 ± 0.03 ^a^	0.65 ± 0.01 ^b^		−1.44 ± 0.02 ^f^	114.34 ± 0.12 ^d^	1.58 ± 0.01 ^e^		0.02 ± 0.00 ^b^
		SLE	87.50 ± 0.03 ^c^	−1.06 ± 0.04 ^d^		3.09 ± 0.06 ^b^	108.14 ± 0.11 ^g^	3.27 ± 0.08 ^b^		0.04 ± 0.01 ^b^
*P. ostreatus*		MHG	90.07 ± 0.02 ^a^	0.71 ± 0.02 ^a^		−1.59 ± 0.02 ^g^	114.11 ± 0.10 ^d^	1.74 ± 0.01 ^c^		0.02 ± 0.01 ^b^
		SLE	87.67 ± 0.05 ^c^	0.23 ± 0.04 ^c^		−0.67 ± 0.02 ^d^	109.14 ± 0.13 ^f^	0.71 ± 0.02 ^i^		0.01 ± 0.00 ^b^
*B. rapa*		MHG	90.04 ± 0.02 ^a^	0.71 ± 0.02 ^a^		−1.65 ± 0.01 ^g^	113.24 ± 0.10 ^e^	1.80 ± 0.01 ^c^		0.02 ± 0.01 ^b^
		SLE	83.99 ± 0.07 ^d^	−6.45 ± 0.05 ^e^		13.38 ± 0.07 ^a^	115.77 ± 0.16 ^c^	14.85 ± 0.15 ^a^		0.18 ± 0.03 ^a^
*Q. robur*	Whole	MHG	90.15 ± 0.02 ^a^	0.66 ± 0.02 ^a,b^		−1.23 ± 0.01 ^e^	118.22 ± 0.10 ^b^	1.40 ± 0.02 ^f^		0.02 ± 0.01 ^b^
		SLE	90.31 ± 0.04 ^a^	0.68 ± 0.01 ^a^		−1.50 ± 0.07 ^f,g^	114.36 ± 0.14 ^d^	1.65 ± 0.01 ^d^		0.02 ± 0.00 ^b^
	Into quarters	MHG	89.25 ± 0.03 ^b^	0.62 ± 0.04 ^b^		−0.68 ± 0.04 ^d^	132.06 ± 0.13 ^a^	0.92 ± 0.01 ^h^		0.01 ± 0.00 ^b^
		SLE	89.64 ± 0.05 ^b^	0.29 ± 0.03 ^c^		1.27 ± 0.08 ^c^	67.21 ± 0.15 ^h^	1.33 ± 0.01 ^g^		0.01 ± 0.00 ^b^
**MHG**			Total Color Difference (ΔE*)				Hue Difference (ΔH*)			
			SLE	*C. scoparius*		5.50	SLE	*C. scoparius*		4.54
				*P. ostreatus*		2.61		*P. ostreatus*		0.13
				*B. rapa*		17.71		*B. rapa*		10.34
				*Q. robur*	Whole	0.31		*Q. robur*	Whole	0.10
					Into quarters	2.02			Into quarters	1.93

Data are given as mean ± standard deviation. Data values in a column with different superscript letters are statically different (*p* ≤ 0.05).

## Supplementary Materials

The following are available online. Figure S1: Effect of the microwave irradiation power during microwave hydrodiffusion and gravity (MHG) extraction at 100 W (a) aqueous phase collected and (b) extraction yield of total solid of aqueous phase recovered of the different supplied raw materials. Values of the required energy consumption required are also summarized for comparative purposes; Figure S2: Total solid content of the sun cream formulated with distilled or thermal waters and with selected MHG extracts from defrosted *C. scoparius* flowers (grey bars), defrosted fruiting bodies of *P. ostreatus* mushrooms (bars with vertical lines), defrosted *B. rapa* leaves (texture bars) and quarters of *Q. robur* acorns (zigzag bars). For comparative purpose, data of sun cream formulated with BHT (bars with horizontal lines), (±)-*α*-tocopherol (dotted bars) and water control (squared bars) are also showed; Figure S3: Evolution of pH values during accelerated oxidation determination of the sun cream formulated with distilled or thermal waters and with selected MHG extracts from defrosted *C. scoparius* flowers (dark grey bars), defrosted fruiting bodies of *P. ostreatus* mushrooms (light grey bars), defrosted *B. rapa* leaves (grey bars) and quarters of *Q. robur* acorns (bars with vertical lines) and BHT (bars with horizontal lines), (±)-*α*-tocopherol (dotted bars) and water control (squared bars) with (a) distilled water, (b) thermal spring water 1, (c) thermal spring water 2 and (d) thermal spring water 3; Figure S4: Evolution of thiobarbituric acid reactive substances (TBARS) during accelerated oxidation determination of the sun cream formulated with distilled or thermal waters and with selected MHG extracts from defrosted *C. scoparius* flowers (dark grey bars), defrosted fruiting bodies of *P. ostreatus* mushrooms (light grey bars), defrosted *B. rapa* leaves (grey bars) and quarters of *Q. robur* acorns (bars with vertical lines) and BHT (bars with horizontal lines), (±)-*α*-tocopherol (dotted bars) and water control (squared bars) with (a) distilled water, (b) thermal spring water 1, (c) thermal spring water 2 and (d) thermal spring water 3.

## References

[1] Kozłowska J., Prus W., Stachowiak N. (2019). Microparticles based on natural and synthetic polymers for cosmetic applications. Int. J. Boil. Macromol..

[2] Rafiee Z., Nejatian M., Daeihamed M., Jafari S.M. (2019). Application of curcumin-loaded nanocarriers for food, drug and cosmetic purposes. Trends Food Sci. Technol..

[3] An J., Lee I., Yi Y. (2019). The Thermal Effects of Water Immersion on Health Outcomes: An Integrative Review. Int. J. Environ. Res. Public Health.

[4] Matsumoto S. (2018). Evaluation of the Role of Balneotherapy in Rehabilitation Medicine. J. Nippon. Med Sch..

[5] Ferreira E.B., Vasques C.I., Gadia R., Chan R.J., Guerra E.N.S., Mezzomo L.A., De Luca Canto G., dos Reis P.E.D. (2017). Topical interventions to prevent acute radiation dermatitis in head and neck cancer patients: A systematic review. Support. Care Cancer.

[6] Maarouf M., Saberian C., Lio P.A., Shi V.Y. (2018). Head-and-neck dermatitis: Diagnostic difficulties and management pearls. Pediatr. Dermatol..

[7] Zeichner J., Seite S. (2018). From probiotic to prebiotic using thermal spring water. J. Drugs Dermatol..

[8] Afonso T., Moresco R., Uarrota V.G., Navarro B.B., Nunes E.D.C., Maraschin M., Rocha M. (2017). UV-Vis and CIELAB Based Chemometric Characterization of Manihot esculenta Carotenoid Contents. J. Integr. Bioinform..

[9] Morone J., Alfeus A., Vasconcelos V., Martins R. (2019). Revealing the potential of cyanobacteria in cosmetics and cosmeceuticals—A new bioactive approach. Algal Res..

[10] Wongwad E., Pingyod C., Saesong T., Waranuch N., Wisuitiprot W., Sritularak B., Temkitthawon P., Ingkaninan K. (2019). Assessment of the bioactive components, antioxidant, antiglycation and anti-inflammatory properties of Aquilaria crassna Pierre ex Lecomte leaves. Ind. Crops Prod..

[11] González N., Ribeiro D., Fernandes E., Nogueira D.R., Conde E., Moure A., Vinardell M.P., Mitjans M., Domínguez H. (2013). Potential use of Cytisus scoparius extracts in topical applications for skin protection against oxidative damage. J. Photochem. Photobiol. B Boil..

[12] Lores M., Pajaro M., Alvarez-Casas M., Domínguez J., Garcia-Jares C. (2015). Use of ethyl lactate to extract bioactive compounds from Cytisus scoparius: Comparison of pressurized liquid extraction and medium scale ambient temperature systems. Talanta.

[13] Taofiq O., Heleno S.A., Calhelha R.C., Fernandes I.P., Alves M.J., Barros L., González-Paramás A.M., Ferreira I.C., Barreiro M.F. (2018). Mushroom-based cosmeceutical ingredients: Microencapsulation and in vitro release profile. Ind. Crops Prod..

[14] Taofiq O., Rodrigues F., Barros L., Barreiro M.F., Ferreira I.C., Oliveira M.B.P. (2019). Mushroom ethanolic extracts as cosmeceuticals ingredients: Safety and ex vivo skin permeation studies. Food Chem. Toxicol..

[15] Sena L.M., Zappelli C., Apone F., Barbulova A., Tito A., Leone A., Olivoero T., Ferracane R., Fogliano V., Colucci G. (2018). Brassica rapa hairy root extracts promote skin depigmentation by modulating melanin production and distribution. J. Cosmet. Dermatol..

[16] Subramanian V., Sahithya D. (2016). Preliminary Screening of Selected Plant Extracts for Anti Tyrosinase Activity. J. Nat. Remedies.

[17] Laddha A.P., Kulkarni Y.A. (2019). Tannins and vascular complications of Diabetes: An update. Phytomedicine.

[18] Sillero L., Prado R., Andrés M.A., Labidi J. (2019). Characterisation of bark of six species from mixed Atlantic forest. Ind. Crops Prod..

[19] Chemat F., Abert-Vian M., Fabiano-Tixier A.S., Strube J., Uhlenbrock L., Gunjevic V., Cravotto G. (2019). Green extraction of natural products. Origins, current status, and future challenges. Trends Analyt. Chem..

[20] Razzaghi S.E., Arabhosseini A., Turk M., Soubrat T., Cendres A., Kianmehr M.H., Perino S., Chemat F. (2019). Operational efficiencies of six microwave based extraction methods for orange peel oil. J. Food Eng..

[21] Reyes-Ocampo I., Córdova-Aguilar M.S., Guzmán G., Blancas-Cabrera A., Ascanio G. (2019). Solvent-free mechanical extraction of Opuntia ficus-indica mucilage. J. Food Process. Eng..

[22] Cendres A., Hoerlé M., Chemat F., Renard C.M. (2014). Different compounds are extracted with different time courses from fruits during microwave hydrodiffusion: Examples and possible causes. Food Chem..

[23] Rodríguez-Seoane P., Díaz-Reinoso B., Muñoz G.-, Portela C.F.D.A., Domínguez H. (2019). Innovative technologies for the extraction of saccharidic and phenolic fractions from *Pleurotus eryngii*. LWT.

[24] Benmoussa H., Elfalleh W., He S., Romdhane M., Benhamou A., Chawech R. (2018). Microwave hydrodiffusion and gravity for rapid extraction of essential oil from Tunisian cumin (*Cuminum cyminum* L.) seeds: Optimization by response surface methodology. Ind. Crop. Prod..

[25] Darvishi H., Azadbakht M., Rezaeiasl A., Farhang A. (2013). Drying characteristics of sardine fish dried with microwave heating. J. Saudi Soc. Agric. Sci..

[26] Singleton V.L., Rossi J.A. (1965). Colorimety of total phenolics with phosphomolybdic-phosphotungstic acid reagents. Am. J. Enol Viticult..

[27] Re R., Pellegrini N., Proteggente A., Pannala A., Yang M., Rice-Evans C. (1999). Antioxidant activity applying an improved ABTS radical cation decolorization assay. Free. Radic. Boil. Med..

[28] Benzie I.F., Strain J. (1996). The Ferric Reducing Ability of Plasma (FRAP) as a Measure of “Antioxidant Power”: The FRAP Assay. Anal. Biochem..

[29] Gadow A., Joubert E., Hansmann C. (1997). Comparison of the antioxidant activity of rooibos tea (*Aspalathus linearis*) with green, oolong and black tea. Food Chem..

[30] Khosa M.K., Chatha S.A.S., Hussain A.I., Zia K.M., Riaz H., Aslam K. (2011). Spectrophotometric quantification of antioxidant phytochemicals in juices from four different varieties of Citrus limon indigenous to Pakistan. J. Chem. Soc. Pak..

[31] Liyanaarachchi G.D., Samarasekera J.K.R.R., Mahanama K.R.R., Hemalal K.D.P. (2018). Tyrosinase, elastase, hyaluronidase, inhibitory and antioxidant activity of Sri Lankan medicinal plants for novel cosmeceuticals. Ind. Crops Prod..

[32] Chiari M., Joray M., Ruiz G., Palacios S., Carpinella M. (2010). Tyrosinase inhibitory activity of native plants from central Argentina: Isolation of an active principle from *Lithrea molleoides*. Food Chem..

[33] Balboa E.M., Rivas S., Moure A., Domínguez H., Parajó J.C. (2013). Simultaneous Extraction and Depolymerization of Fucoidan from *Sargassum muticum* in Aqueous Media. Mar. Drugs.

[34] Garrote G., Domínguez H., Parajó J.C. (2001). Manufacture of xylose-based fermentation media from corncobs by posthydrolysis of autohydrolysis liquors. Appl. Biochem. Biotechnol..

[35] Bradford M.M. (1976). A rapid and sensitive method for the quantitation of microgram quantities of protein utilizing the principle of protein-dye binding. Anal. Biochem..

[36] Kamal I. Identification and Extraction Kinetics of Lipids Using UV Spectrophotometry. Technical Report. https://www.researchgate.net/publication/319128233_Identification_and_Extraction_Kinetics_of_Lipids_Using_UV_Spectrophotometry.

[37] Kaur C.D., Saraf S. (2010). In vitro sun protection factor determination of herbal oils used in cosmetics. Pharmacognosy Res..

[38] Mansur J.S., Breder M.N.R., Mansur M.C.A., Azulay R.D. (1986). Determinação do fator de proteção solar por espectrofotometría. An. Bras. Dermatol..

[39] Sayre R.M., Agin P.P., Levee G.J., Marlowe E. (1979). A Comparison Of In Vivo And In Vitro Testing of Sunscreening Formulas. Photochem. Photobiol..

[40] Balboa E.M., Soto M.L., Nogueira D.R., González-López N., Conde E., Moure A., Vinardell M.P., Mitjans M., Domínguez H. (2014). Potential of antioxidant extracts produced by aqueous processing of renewable resources for the formulation of cosmetics. Ind. Crops Prod..

[41] Dutra E.A., Oliveira D.A.G.D.C., Kedor-Hackmann E.R.M., Santoro M.I.R.M. (2004). Determination of sun protection factor (SPF) of sunscreens by ultraviolet spectrophotometry. Revista Brasileira de Ciências Farmacêuticas.

[42] Scheffler S.L., Wang X., Huang L., Gonzalez F.S.-M., Yao Y. (2010). Phytoglycogen Octenyl Succinate, an Amphiphilic Carbohydrate Nanoparticle, and ε-Polylysine To Improve Lipid Oxidative Stability of Emulsions. J. Agric. Food Chem..

[43] Chemat F., Vian M., Franco V. (2008). Microwave Hydrodiffusion for Isolation of Natural Products. European Patent.

[44] Makanjuola S.A. (2017). Influence of particle size and extraction solvent on antioxidant properties of extracts of tea, ginger, and tea-ginger blend. Food Sci. Nutr..

[45] López-Hortas L., Gely M., Falqué E., Domínguez H., Torres M.D. (2019). Alternative environmental friendly process for dehydration of edible *Undaria pinnatifida* brown seaweed by microwave hydrodiffusion and gravity. J. Food Eng..

[46] Ekezie F.-G.C., Sun D.-W., Cheng J.-H. (2017). Acceleration of microwave-assisted extraction processes of food components by integrating technologies and applying emerging solvents: A review of latest developments. Trends Food Sci. Technol..

[47] Iqbal S., Younas U., Chan K.W., Saeed Z., Shaheen M.A., Akhtar N., Majeed A. (2013). Growth and antioxidant response of *Brassica rapa var. rapa* L. (turnip) irrigated with different compositions of paper and board mill (PBM) effluent. Chemosphere.

[48] Rakic S., Petrović S., Kukić J., Jadranin M., Tesevic V., Povrenovic D., Siler-Marinkovic S. (2007). Influence of thermal treatment on phenolic compounds and antioxidant properties of oak acorns from Serbia. Food Chem..

[49] Cipolatti E.P., Remedi R.D., Sá C.D.S., Rodrigues A.B., Ramos J.M.G., Burkert C.A.V., Furlong E.B., Burkert J.F.D.M. (2019). Use of agroindustrial byproducts as substrate for production of carotenoids with antioxidant potential by wild yeasts. Biocatal. Agric. Biotechnol..

[50] Neha P., Pandey-Rai S. (2014). Biochemical activity and therapeutic role of antioxidants in plants and humans. Plants as a Source of Natural Antioxidants.

[51] Frede K., Schreiner M., Baldermann S. (2019). Light quality-induced changes of carotenoid composition in pak choi Brassica rapa ssp. chinensis. J. Photochem. Photobiol. B Boil..

[52] Bessa L.C., Ferreira M.C., Rodrigues C.E., Batista E.A., Meirelles A.J. (2017). Simulation and process design of continuous countercurrent ethanolic extraction of rice bran oil. J. Food Eng..

[53] Page J.C., Arruda N.P., Freitas S.P. (2017). Crude ethanolic extract from spent coffee grounds: Volatile and functional properties. Waste Manag..

[54] Thavarajah D., Thavarajah P., Abare A., Basnagala S., Lacher C., Smith P., Combs G.F. (2016). Mineral micronutrient and prebiotic carbohydrate profiles of USA-grown kale (*Brassica oleracea* L. var. acephala). J. Food Compos. Anal..

[55] Rachmawati H., Sundari S., Nabila N., Tandrasasmita O.M., Amalia R., Siahaan T.J., Tjandrawinata R.R., Ismaya W.T. (2019). Orf239342 from the mushroom *Agaricus bisporus* is a mannose binding protein. Biochem. Biophys. Res. Commun..

[56] Zhang M., Liu Y., Song C., Ning J., Cui Z. (2019). Characterization and functional analysis of a novel mannose-binding lectin from the swimming crab *Portunus trituberculatus*. Fish Shellfish. Immunol..

[57] Adekunte A., Tiwari B., Cullen P., Scannell A., O’Donnell C. (2010). Effect of sonication on colour, ascorbic acid and yeast inactivation in tomato juice. Food Chem..

[58] Afonso C., Hirano R., Gaspar A., Chagas E., Carvalho R., Silva F., Leonardi G., Lopes P., Silva C., Yoshida C. (2019). Biodegradable antioxidant chitosan films useful as an anti-aging skin mask. Int. J. Boil. Macromol..

[59] Conesa A., Manera F., Brotons J., Fernandez-Zapata J., Simón I., Simón-Grao S., Alfosea-Simón M., Nicolás J.M., Valverde J., García-Sanchez F. (2019). Changes in the content of chlorophylls and carotenoids in the rind of Fino 49 lemons during maturation and their relationship with parameters from the CIELAB color space. Sci. Hortic..

[60] Bom S., Jorge J., Ribeiro H., Marto J. (2019). A step forward on sustainability in the cosmetics industry: A review. J. Clean. Prod..

[61] Chiocchio I., Mandrone M., Sanna C., Maxia A., Tacchini M., Poli F. (2018). Screening of a hundred plant extracts as tyrosinase and elastase inhibitors, two enzymatic targets of cosmetic interest. Ind. Crops Prod..

[62] Delgado-Outeiriño I., Araujo-Nespereira P., Cid-Fernández J., Mejuto J.C., Martínez-Carballo E., Simal-Gandara J. (2009). Behaviour of thermal waters through granite rocks based on residence time and inorganic pattern. J. Hydrol..

[63] Nurjanah, Luthfiyana N., Hidayat T., Nurilmala M., Anwar E. (2019). Utilization of seaweed porridge *Sargassum* sp. and *Eucheuma cottonii* as cosmetic in protecting skin. IOP Conf. Ser. Earth Environ. Sci..

[64] Ospina M., Montaña-Oviedo K., Díaz-Duque Á, Toloza-Daza H., Narváez-Cuenca C.-E. (2019). Utilization of fruit pomace, overripe fruit, and bush pruning residues from Andes berry (*Rubus glaucus* Benth) as antioxidants in an oil in water emulsion. Food Chem..

[65] Pandolsook S., Kupongsak S. (2019). Storage stability of bleached rice bran wax organogels and water-in oil emulsions. J. Food Meas. Charact..

[66] Houlden R.J. Viscosity vs. rheology: Why it is important to formulators. Personal Care 2017. https://www.personalcaremagazine.com/story/24670/viscosity-vs-rheology-why-it-is-important-to-formulators.

[67] Houlden R.J. (2018). The Influence of Rheology on Sunscreen Performance and SPF—Are Highly Thixtotropic Products Not Providing Enough Protection?.

[68] López-Hortas L., Conde E., Falqué E., Domínguez H., Torres M.D. (2019). Preparation of Hydrogels Composed of Bioactive Compounds from Aqueous Phase of Artichoke Obtained by MHG Technique. Food Bioprocess Technol..

[69] López-Hortas L., Conde E., Falqué E., Domínguez H., Torres M. (2019). Recovery of aqueous phase of broccoli obtained by MHG technique for development of hydrogels with antioxidant properties. LWT.

[70] Nooeaid P., Chuysinuan P., Techasakul S. (2017). Alginate/gelatine hydrogels: characterisation and application of antioxidant release. Green Mater..

[71] Pierson J.T., Perino-Issartier S., Ruiz K., Cravotto G., Chemat F. (2016). Laboratory to pilot-scale optimization of microwave hydrodiffusion and gravity: Solvent-free polyphenols extraction from lettuce. Food Chem..

