# Supplementary material for: Microwave Hydrodiffusion and Gravity (MHG) Extraction from Different Raw Materials with Cosmetic Applications
Source: Molecules. 2019 Dec 25;25(1):92. doi: 10.3390/molecules25010092 (PMC6983248; doi:10.3390/molecules25010092)
Supplement: Supplementary file 1 [file molecules-25-00092-s001.pdf]

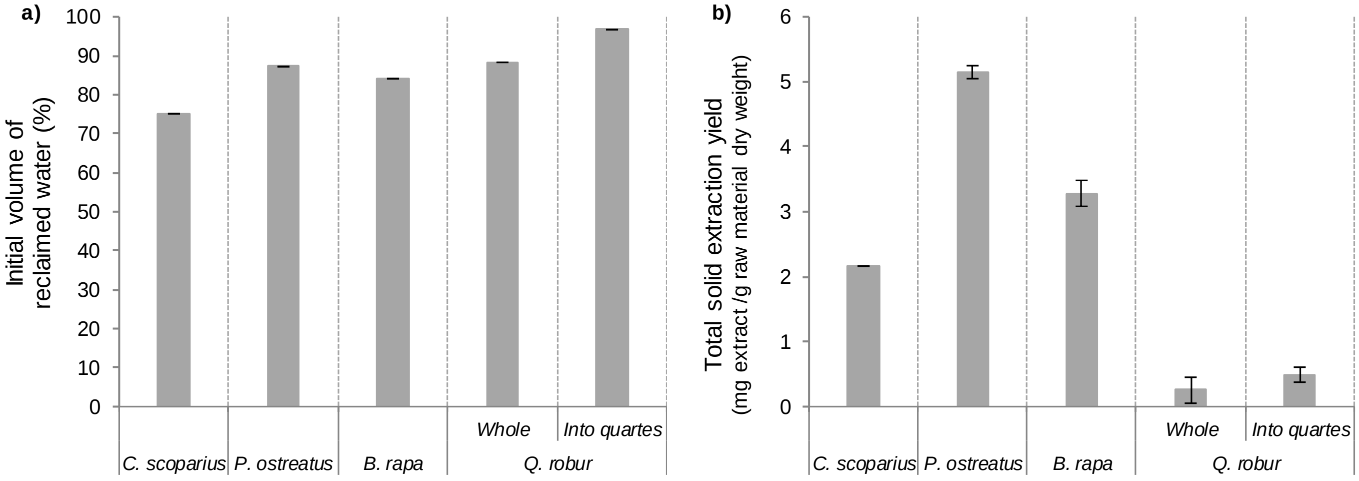

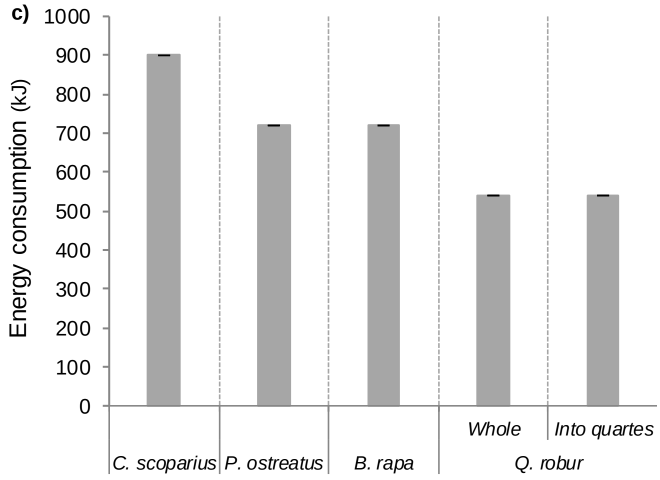

Figure S1: Effect of the microwave irradiation power during microwave hydrodiffusion and gravity (MHG) extraction at 100 W on a) aqueous phase collected and b) extraction yield of total solid of aqueous phase recovered of the different supplied raw materials. Values of the required energy consumption required are also summarized for comparative purposes.

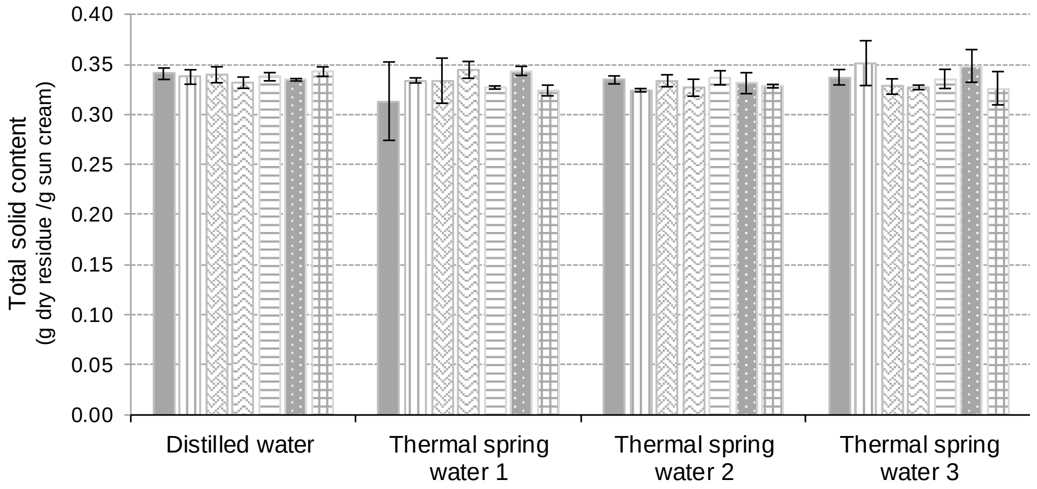

Figure S2: Total solid content of the sun cream formulated with distilled or thermal waters and with selected MHG extracts from defrosted *C. scoparius* flowers (grey bars), defrosted fruiting bodies of *P. ostreatus* mushrooms (bars with vertical lines), defrosted *B. rapa* leaves (texture bars) and quarters of *Q. robur* acorns (zigzag bars). For comparative purpose, data of sun cream formulated with BHT (bars with horizontal lines), (±)-α-tocopherol (dotted bars) and water control (squared bars) are also showed.

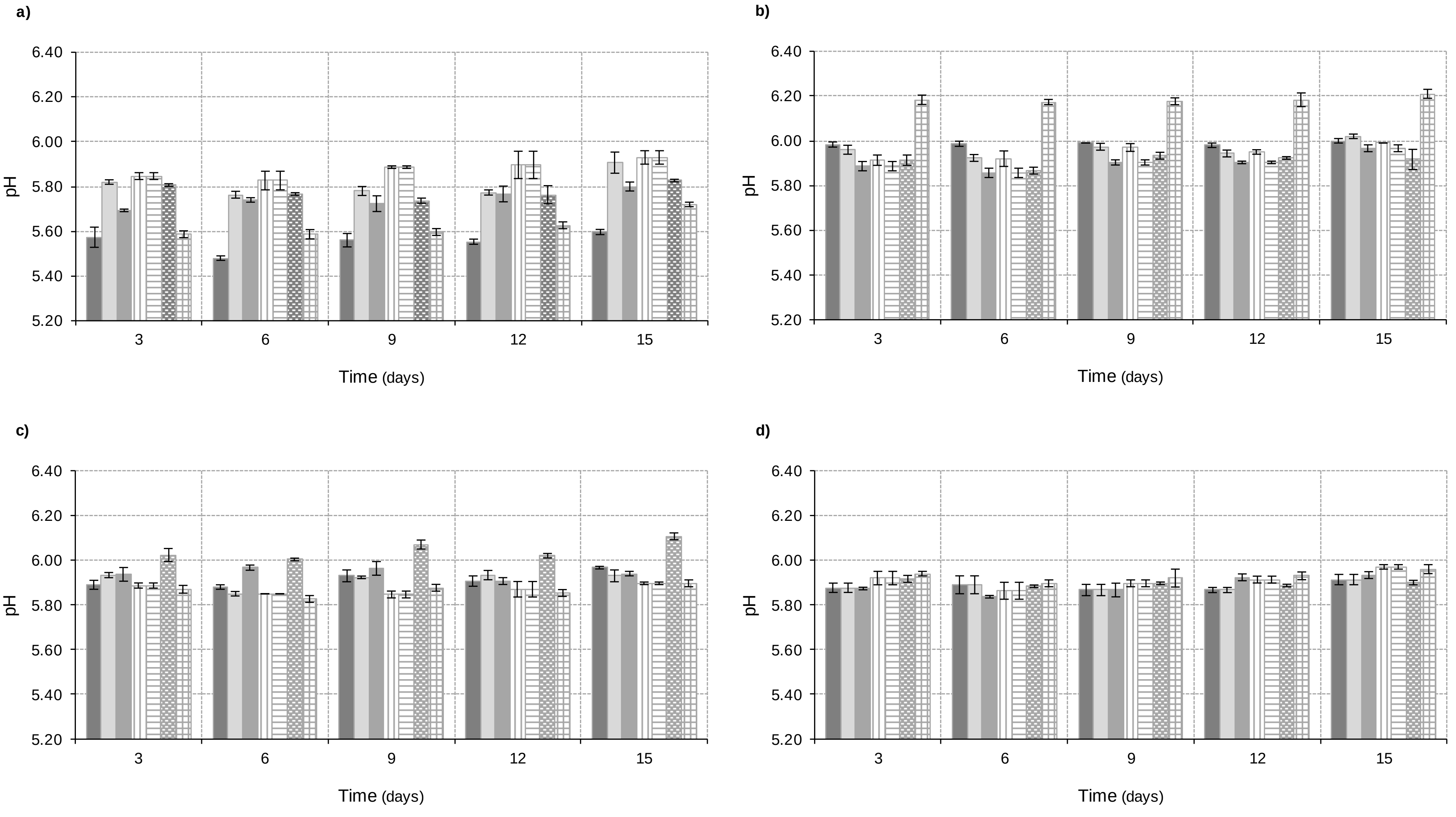

Figure S3: Evolution of pH values during accelerated oxidation determination of the sun cream formulated with distilled or thermal waters and with selected MHG extracts from defrosted *C. scoparius* flowers (dark grey bars), defrosted fruiting bodies of *P. ostreatus* mushrooms (light grey bars), defrosted *B. rapa* leaves (grey bars) and quarters of *Q. robur* acorns (bars with vertical lines) and BHT (bars with horizontal lines), (±)-α-tocopherol (dotted bars) and water control (squared bars) with a) distilled water, b) thermal spring water 1, c) thermal spring water 2 and d) thermal spring water 3.

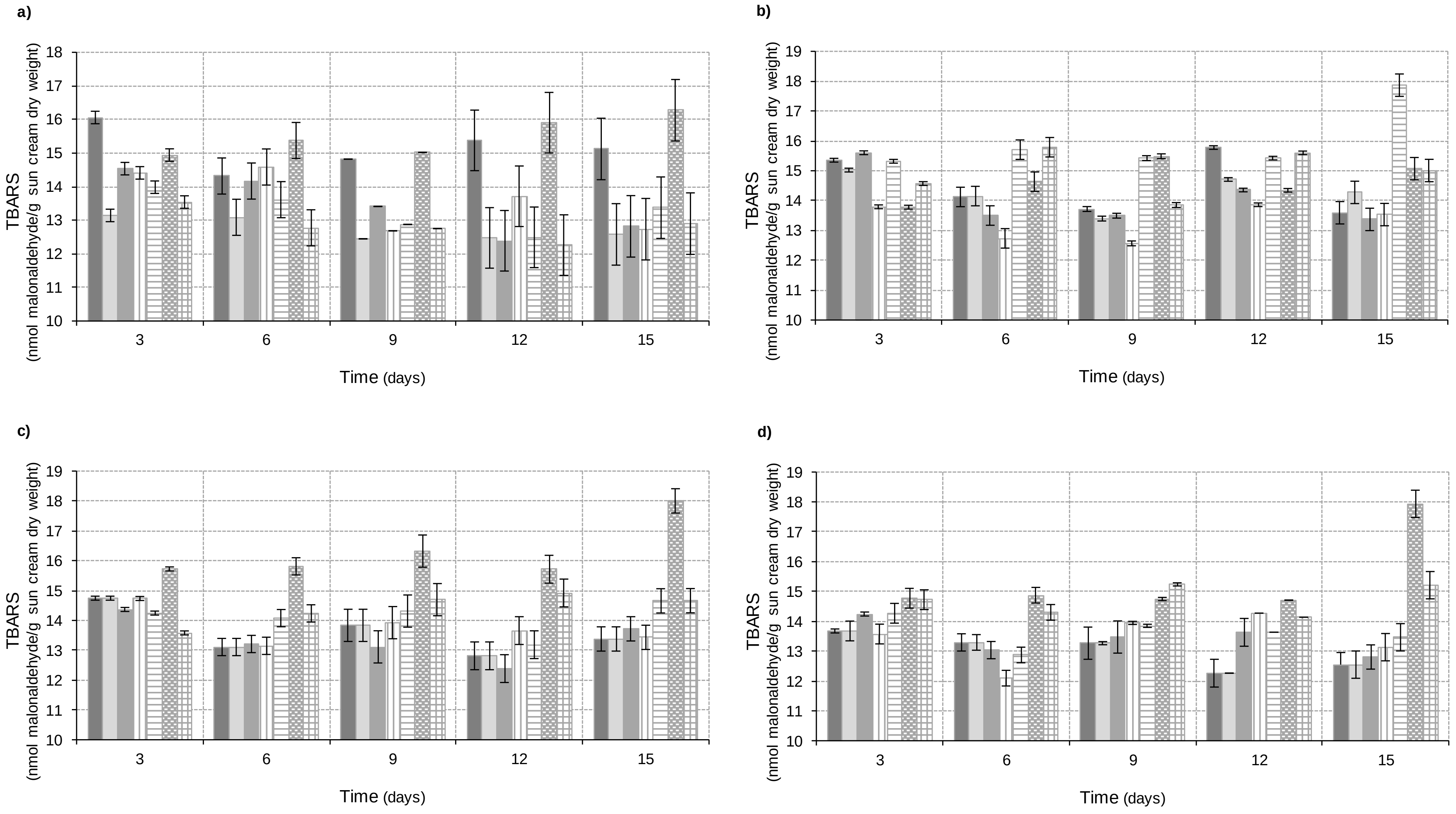

Figure S4: Evolution of thiobarbituric acid reactive substances (TBARS) during accelerated oxidation determination of the sun cream formulated with distilled or thermal waters and with selected MHG extracts from defrosted *C. scoparius* flowers (dark grey bars), defrosted fruiting bodies of *P. ostreatus* mushrooms (light grey bars), defrosted *B. rapa* leaves (grey bars) and quarters of *Q. robur* acorns (bars with vertical lines) and BHT (bars with horizontal lines), (±)-*α*-tocopherol (dotted bars) and water control (squared bars) with a) distilled water, b) thermal spring water 1, c) thermal spring water 2 and d) thermal spring water 3.
